# Supplementary material for: Intradecadal variations in length of day and their correspondence with geomagnetic jerks
Source: Nat Commun. 2020 May 8;11:2273. doi: 10.1038/s41467-020-16109-8 (PMC7210880; doi:10.1038/s41467-020-16109-8)
Supplement: Supplementary file 1 — Supplementary Information [file 41467_2020_16109_MOESM1_ESM.pdf]

Supplementary Information for <Intradecadal variations in length of day and their correspondence with geomagnetic jerks> by Duan et al

Supplementary Figures

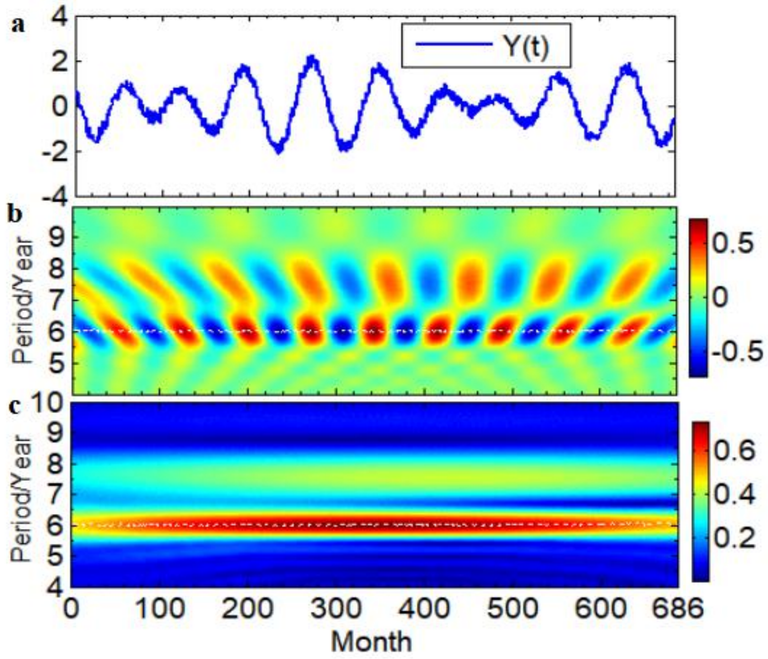

Supplementary Fig. 1 Composite signal $Y(t)$ and its NMWT spectrum. This figure shows that the NMWT method can easily distinguish these two periodic signals (i.e., the 6-year and 7.5-year signals), while the traditional Morlet wavelet transformation cannot do this (see supplementary Fig.4). The white dashed lines in the NMWT spectrum shows the so-called ridge lines[5,12].

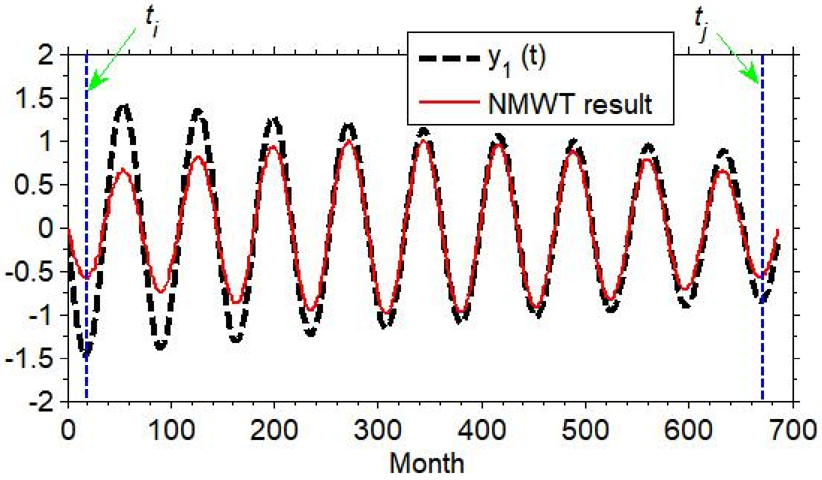

Supplementary Fig. 2 Comparison of the result (the red curve) directly obtained by NMWT without adopting the BEPME strategy and the original simulated signal (the black dashed curve). Here, the edge effect range is calculated by 2 $R_g(a) = 1.643\sigma|a|$ ~ 710 months (here σ =3), which is longer than the data length (i.e., 686 months), hence the target 6 year signal on the whole period is disturbed by the edge effect. This figure shows an

advantage of NMWT method, i.e., the phase information of the target signal directly recovered by NMWT is unbiased, even though the edge effect exists. Using this property, one can search for the correct local extreme points (here, t_i =18 and t_j =671) ,which are closest to the boundaries of the target signal. The data outside these two moments are deleted, and the remaining signals (i.e.19~670 months) are extended by bidirectional mirror-image symmetric extension.

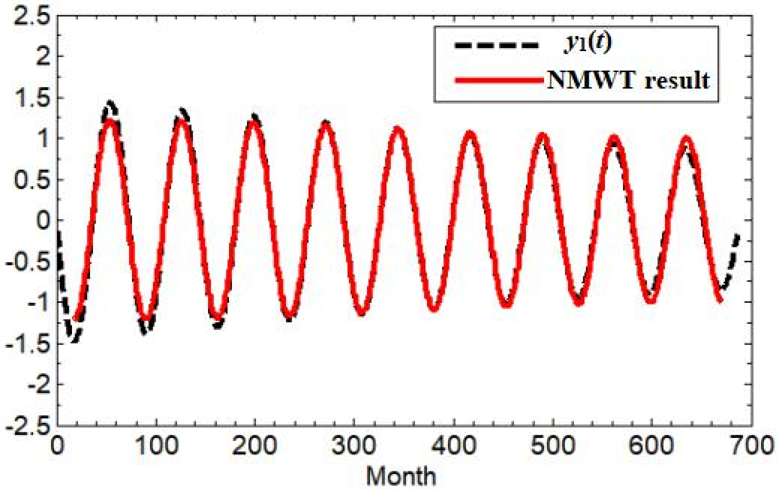

Supplementary Fig. 3 Comparisons of the results obtained by the method (i.e., NMWT+ BEPME) and the original simulated 6-year signal. This figure clearly shows that the edge effects have been significantly repressed comparing with the result shown in supplementary Fig. 2.

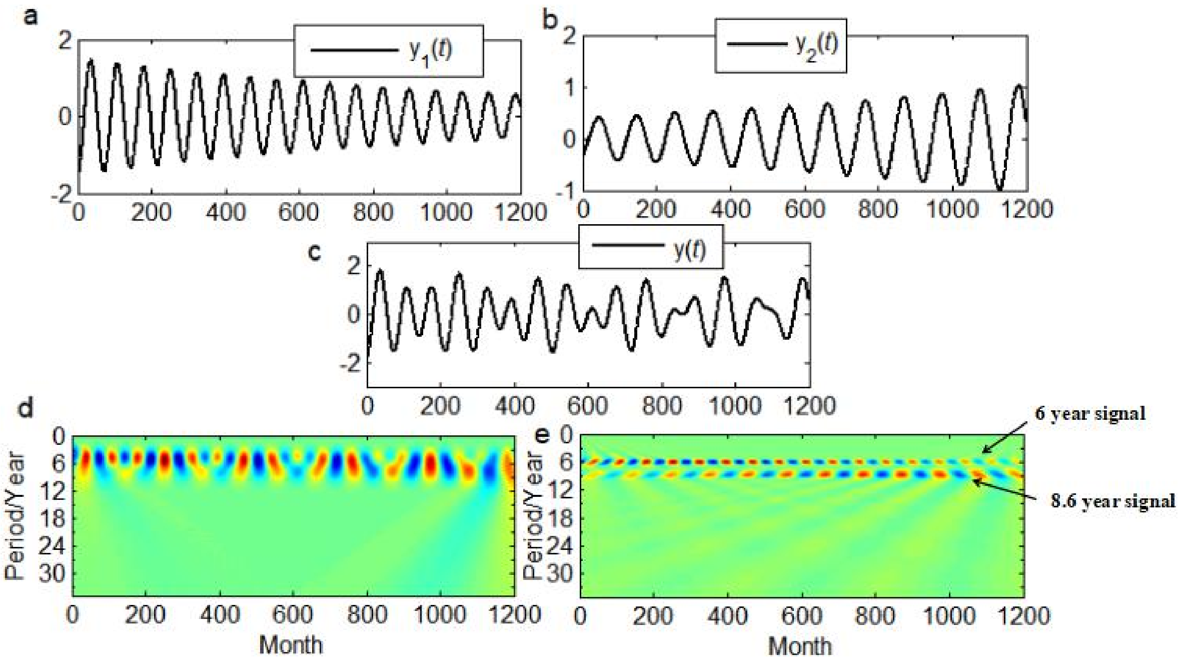

Supplementary Fig.4 Comparison of the NMWT and the TMWT method. **a** shows the 6-year simulated signal; **b** indicates the 8.6-year simulated signal; **c** is the superposition signal of the two harmonic signals; **d** gives the TMWT spectrum, which shows the so-called modulation phenomenon, and it shows that the TMWT method cannot separate the 6 year and 8.6 year signals; **e** presents the NMWT spectrum, where the 6-year and 8.6-year signals are clearly separated.

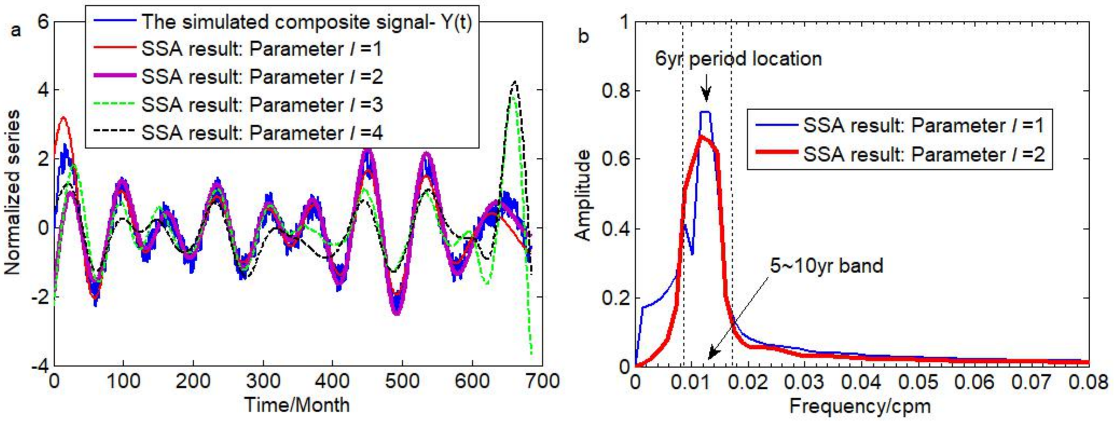

Supplementary Fig.5 Comparison of the SSA result and the original simulated series in both time and frequency domains, here *L*=600.

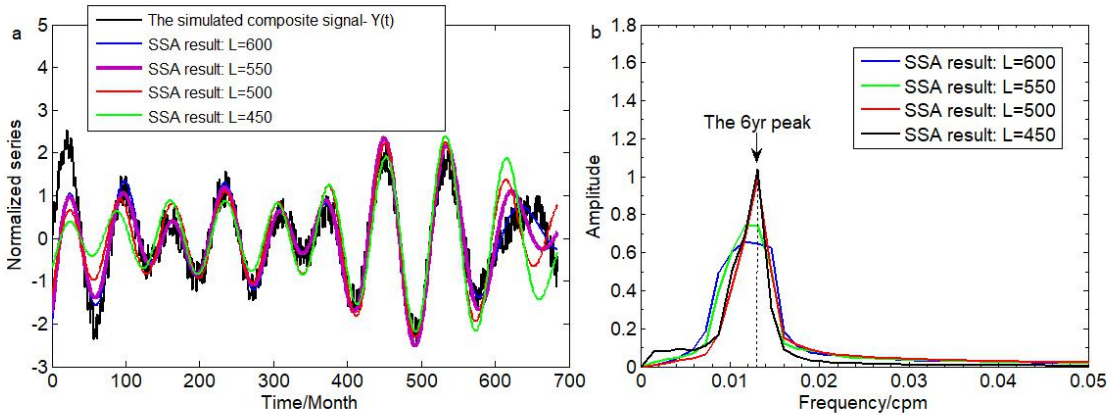

Supplementary Fig.6 Comparison of the SSA result and the original simulated series in both time and frequency domains, here *l*=2.

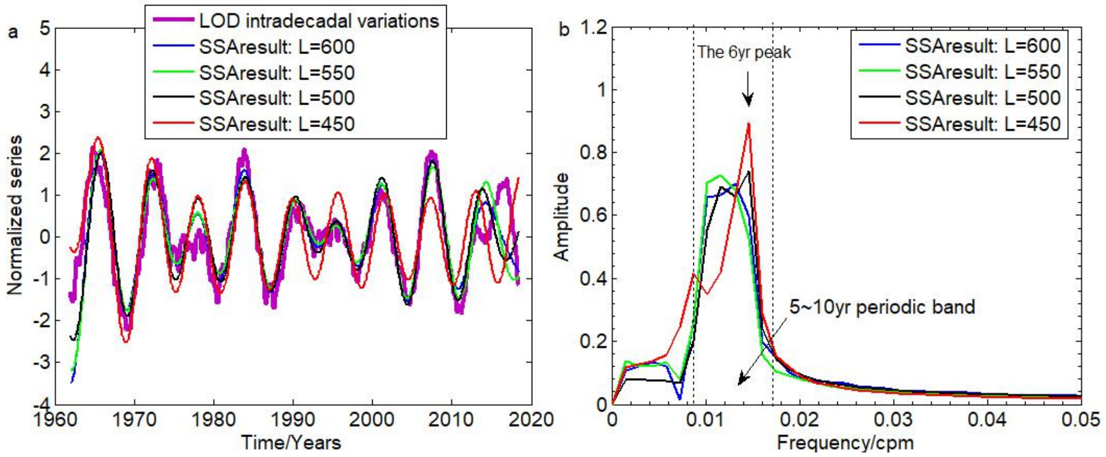

Supplementary Fig.7 Comparison of the SSA result and the original LOD intradecadal variations in both time and frequency domains.

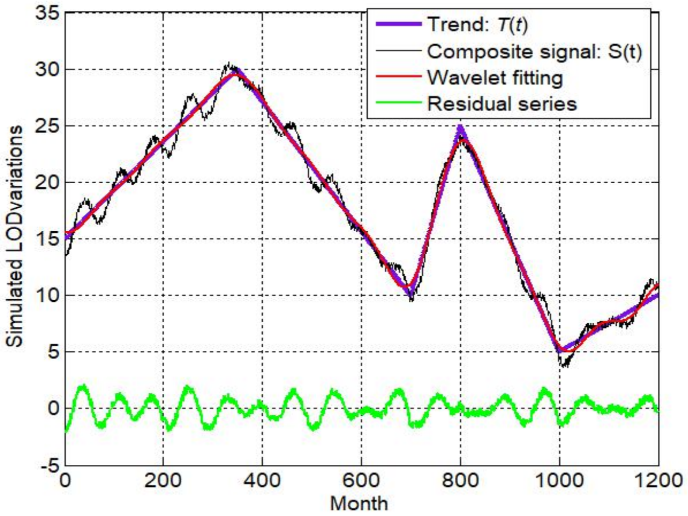

Supplementary Fig.8 The simulated LOD variations. The whole data processing process is the same as that for the actual LOD data. Residual series equals to that $S(t)$ minus the wavelet fitting result.

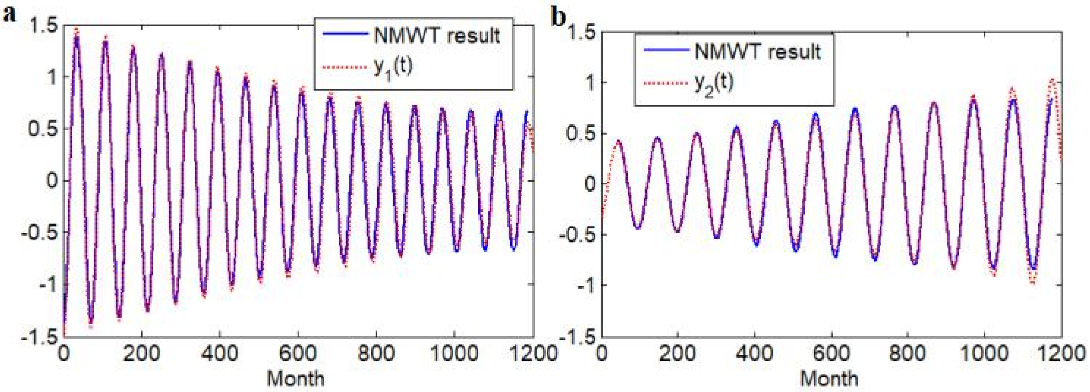

Supplementary Fig. 9 Comparison of the target 6-year and 8.6-year signals recovered by this work with the

original simulation signals

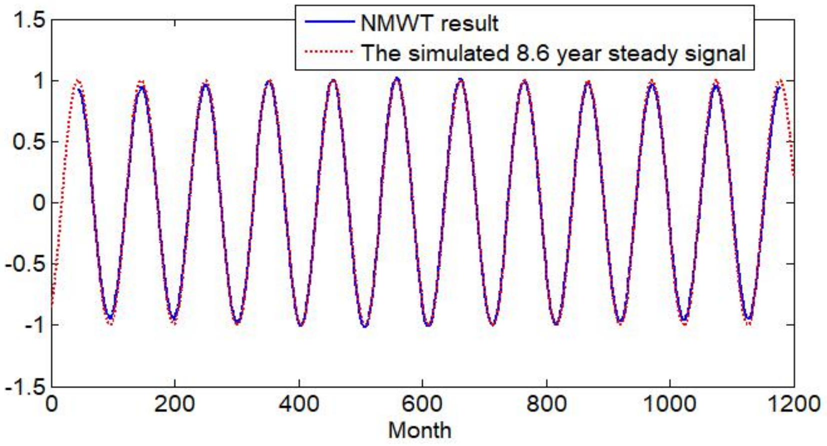

Supplementary Fig. 10 Comparison of the result from the our proposed method and the original simulated 8.6-year pure harmonic signal

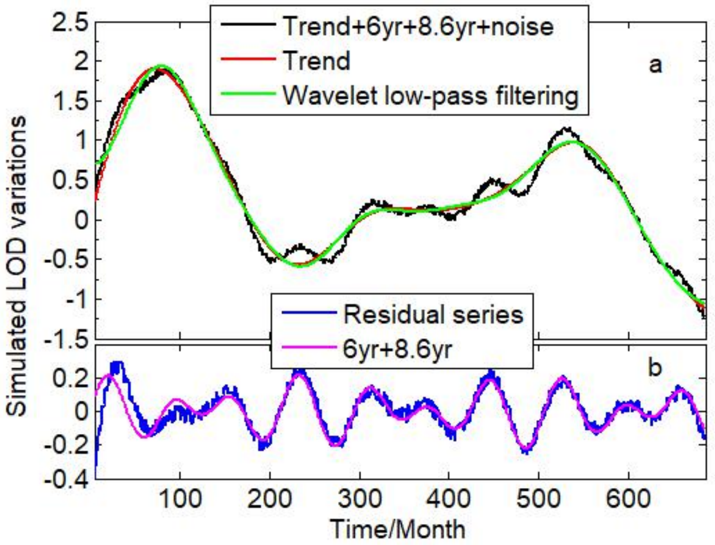

Supplementary Fig.11 **a** shows the simulated LOD variations; **b** indicates the comparison of the residual series and the original simulated intradecadal variations

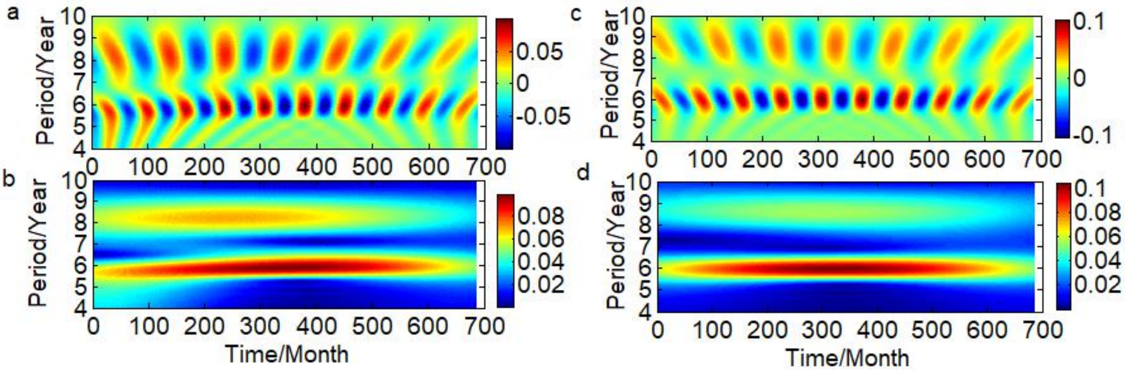

Supplementary Fig.12 a,b show the NMWT time-frequency spectrum results of the residual series after removing the background trend; **c, d** indicate the NMWT time-frequency spectrum results of the simulated *H*(*t*) (i.e., 6yr term+8.6yr term)

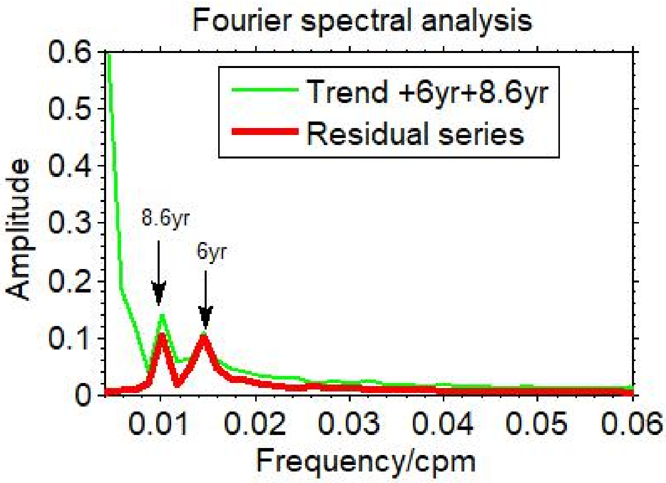

Supplementary Fig. 13 Comparison of the residual series and the simulated signal (Trend+6yr+8.6yr) in frequency domain

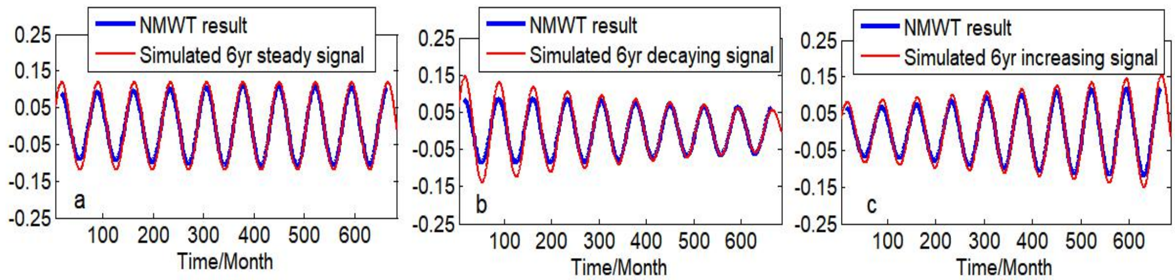

Supplementary Fig.14 Comparison of the the 6yr signals (blue curves) recovered by the NMWT+BEPME method and the original simulated 6yr signals (red curves) in three cases; here, the strategy of avoiding the NMWT edge effect is so-called BEPME approach illustrated in the manuscript.

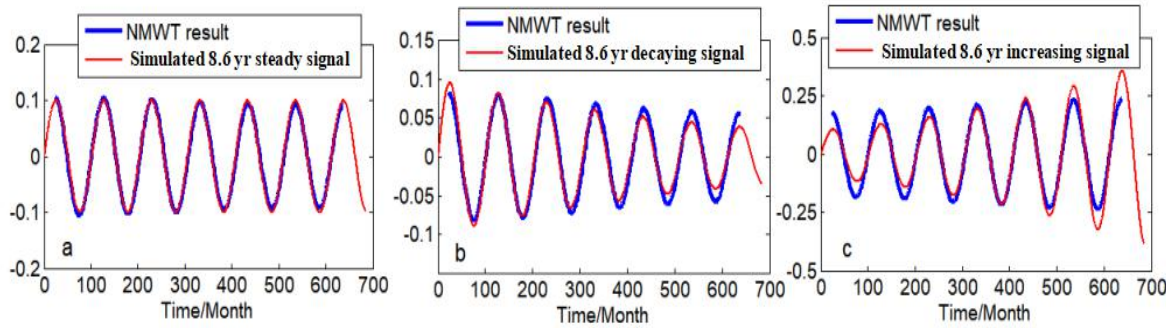

Supplementary Fig.15 Comparison of the the 8.6yr signals (blue curves) recovered by the NMWT+BEPME method and the original simulated 8.6yr signals (red curves) in three cases; here, the strategy of avoiding the NMWT edge effect is so-called BEPME approach illustrated in the manuscript.

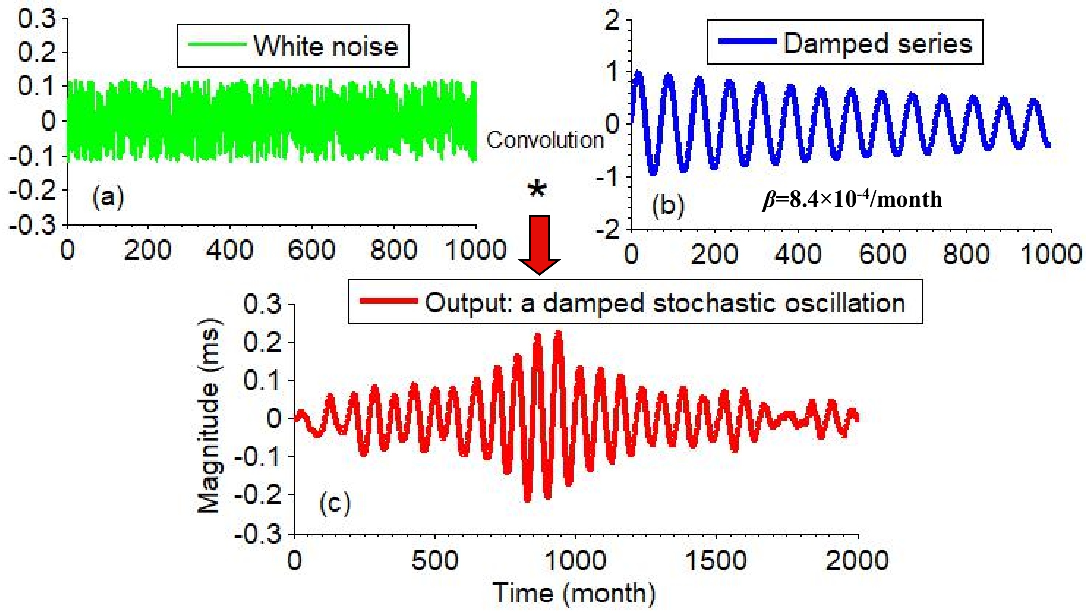

Supplementary Fig.16 **(a)** shows a simulated Gauss white noise stochastic series, which can be see as the input (i.e., $E(t)$) of this convolution operation system expressed by formula (6) of this work; **(b)** displays the damped oscillation function, here β=8.4×10^{-4}/month; **(c)** shows the output series of this convolution operation system, which expresses the 6yr forced damped oscillation series, and this output series depends on the input - $E(t)$.

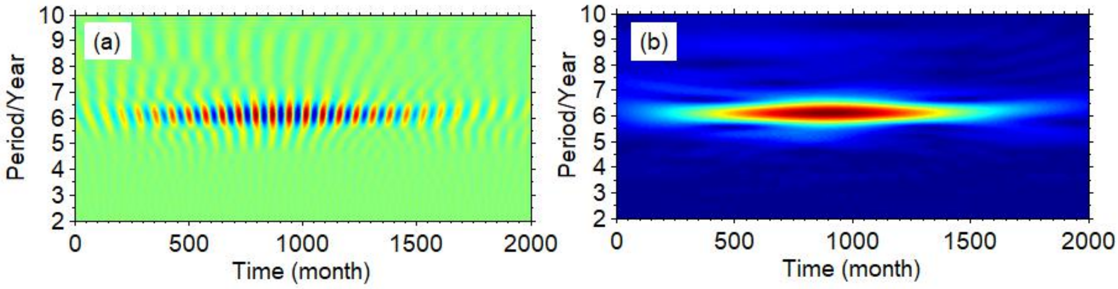

Supplementary Fig.17 The NMWT time-frequency spectrum of the output series

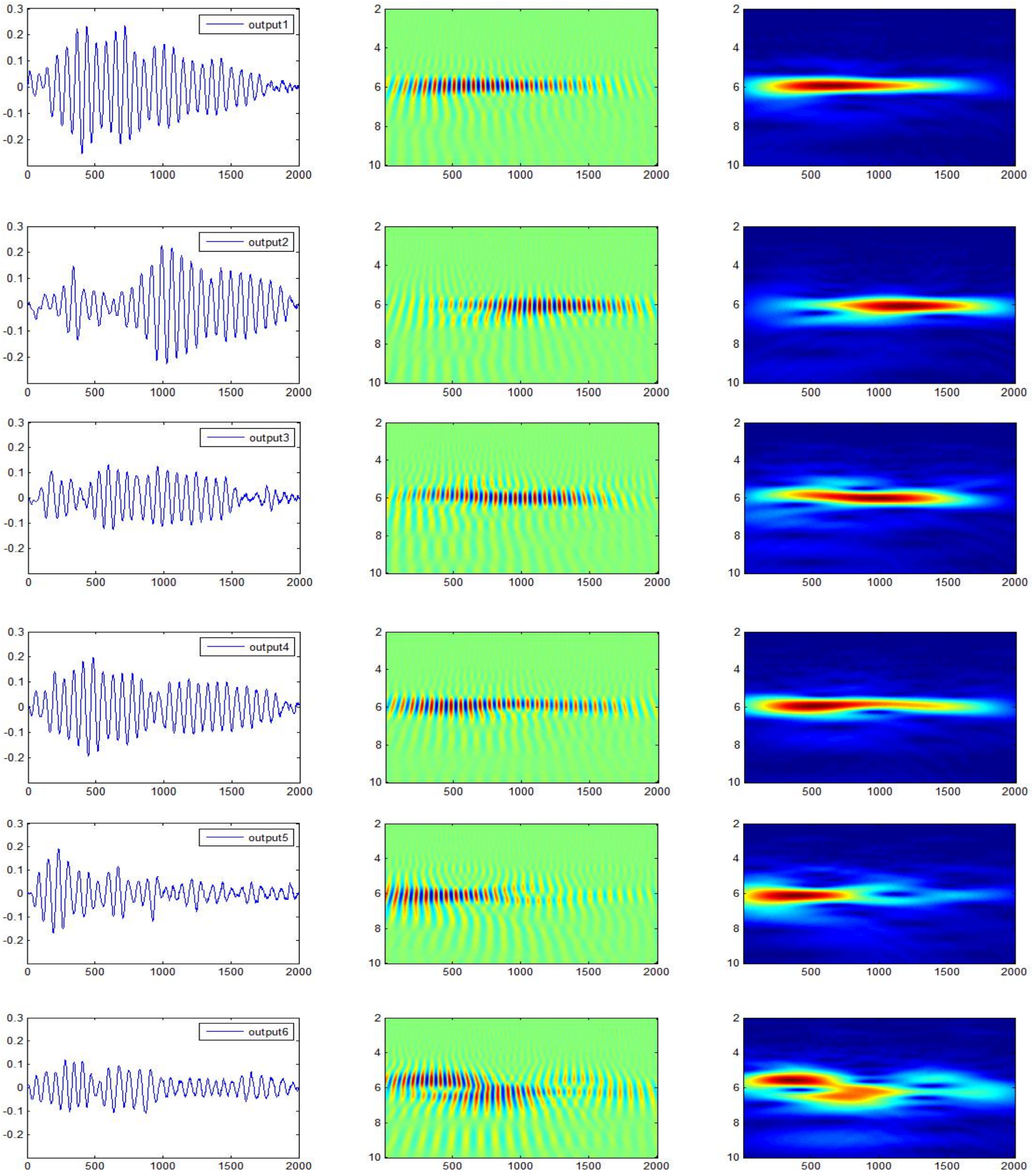

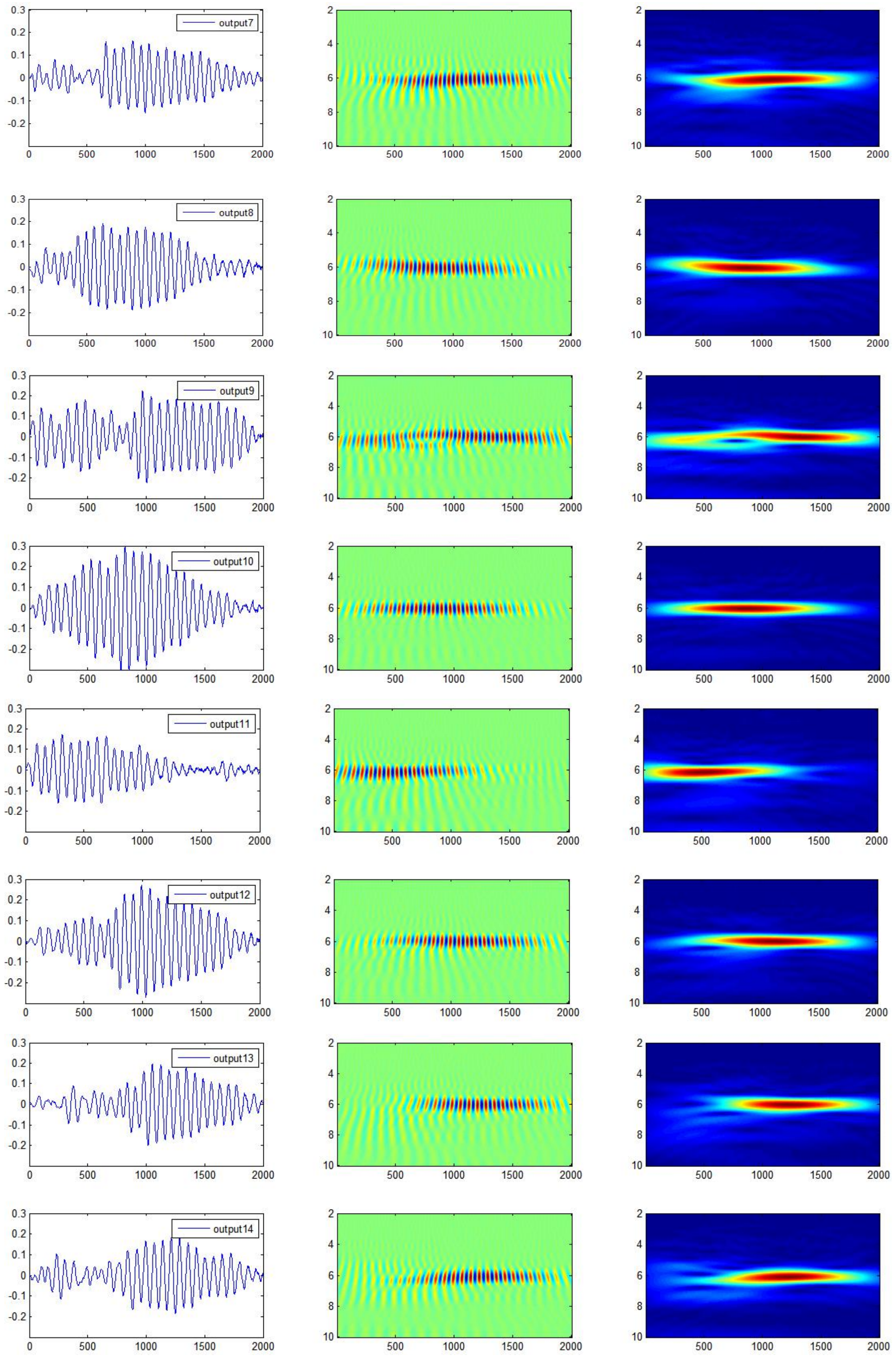
output7
output8
output9
output10
output11
output12
output13
output14

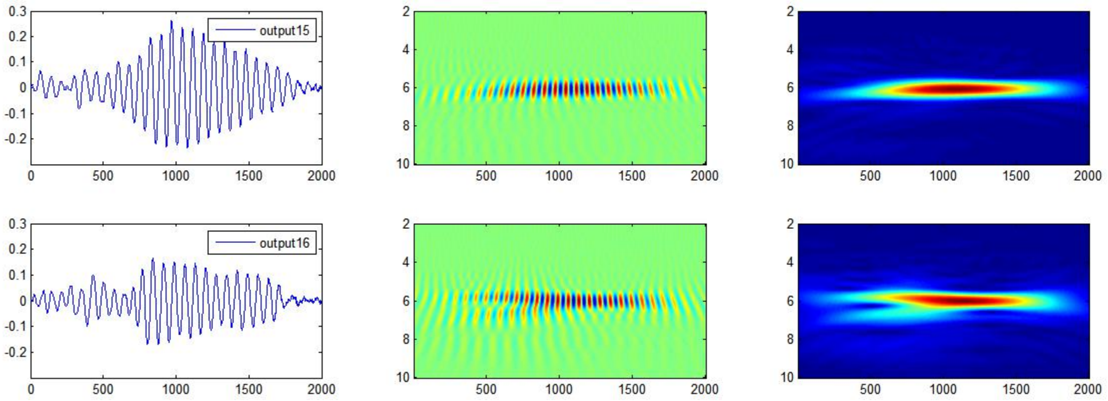

Supplementary Fig. 18 The left columns show the various output series corresponding to the various input series, the middle and the right columns show the NMWT time-frequency spectrum of the output series, where the horizontal ordinates represent the time/month, while the vertical coordinates express the period/year.

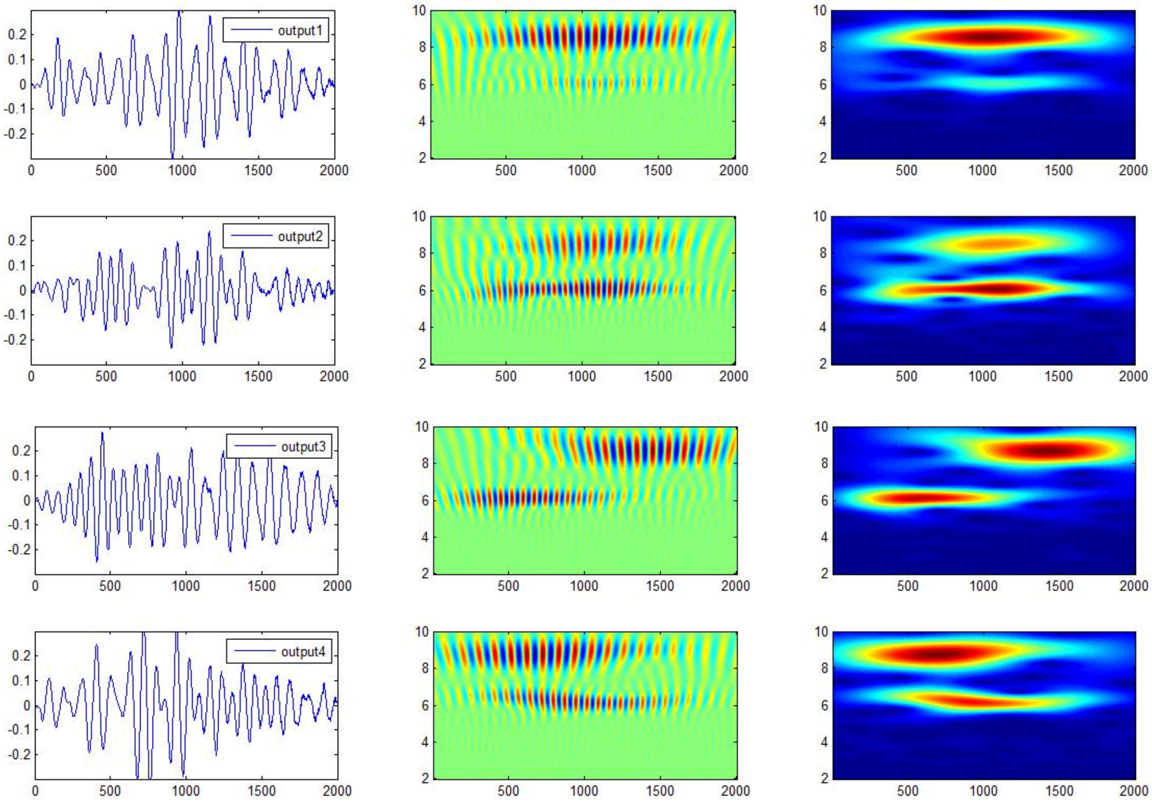

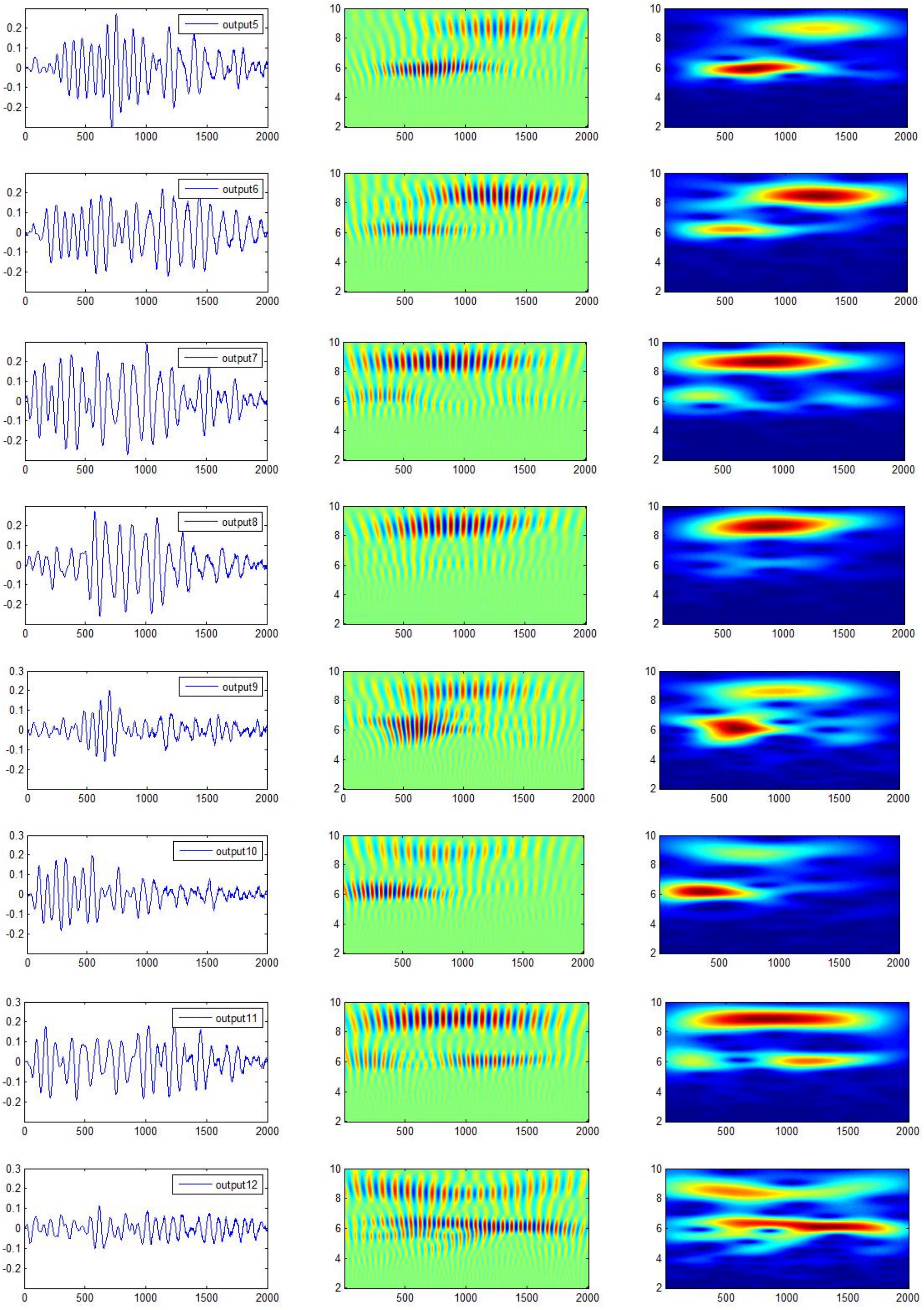
output5
output6
output7
output8
output9
output10
output11
output12

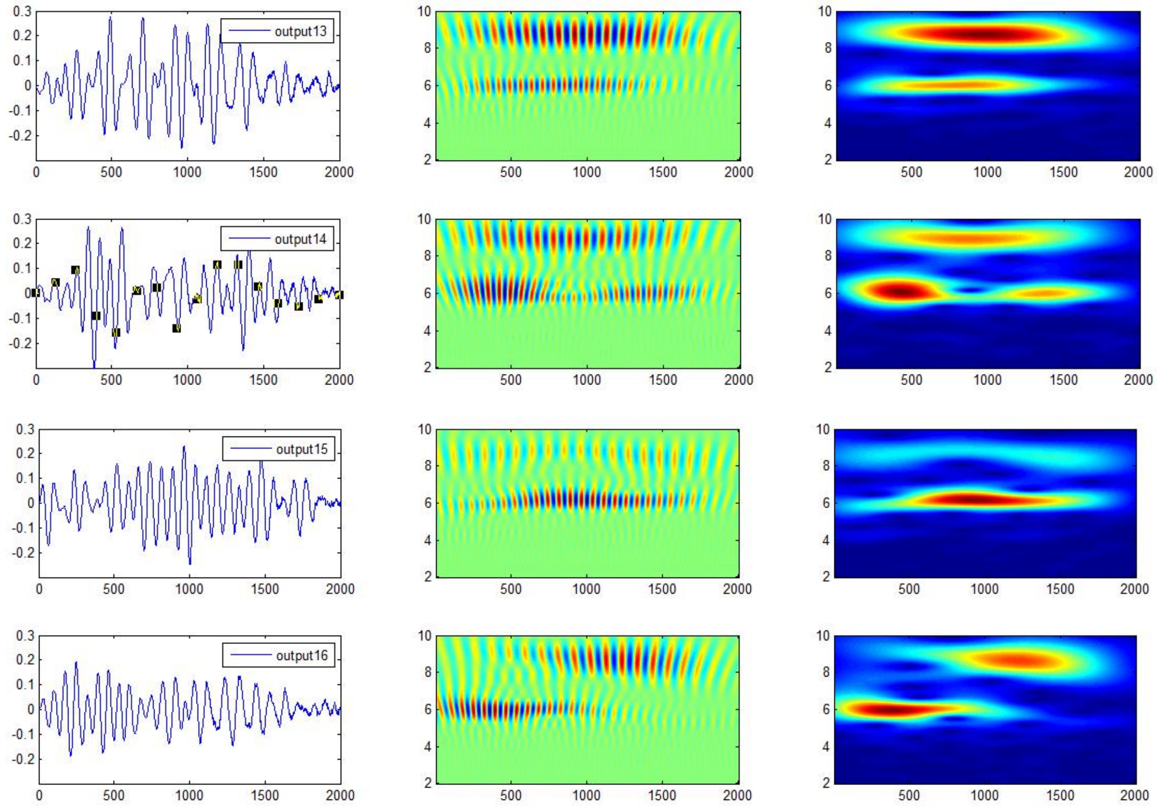

Supplementary Fig.19 The left columns show the various output series (6-year damped stochastic oscillation+8.6-year damped stochastic oscillation), the middle and the right columns show the NMWT time-frequency spectrum of the output series, where the horizontal coordinates represent the time/month, while the vertical coordinates express the period/year.

Supplementary Notes

Up to now, there have been many published works[1-8] to investigate the temporal-varying characteristic of the 6-year oscillation and its mechanism. The normal Morlet wavelet transformation (NMWT) result[5,7] indicated that the 6-year oscillation shows a secular decreasing trend that has never been reported by the other publications. Here, we argue that the other methods, for instance, the traditional Morlet wavelet transformation (TMWT)[3,8-9], the AR-z spectrum[8] and the singular spectrum analysis (SSA), are not suitable for quantitatively recognizing and extracting the target intradecadal temporal-domain variation signals (e.g., the 8.6-year and the 6-year signals) in LOD.

In fact, AR-z spectrum belongs to a frequency-domain analysis method, and thus it is not suitable for analysis of the target 6-year decaying signal in the time domain. In addition, the TMWT method has significant edge effects and poor frequency-resolution, and thus the TMWT method has not the ability to quantitatively display the time-varying features of the target intradecadal harmonic signals in LOD variations. Our recent works[5,7] and the typical simulation examples (e.g., see the Methods and Supplementary Notes 1,3-4) in this work show that the NMWT method is effective to accurately detect the target LOD signals.

Here, in order to solve the controversial issues mentioned above and test whether one method used is suitable for extracting the target intradecadal variations in LOD, we suggest that (at least) a simulation example should be given. However, such kind of the necessary simulation example, to our knowledge, has never been given by other relevant publications to justify the reliability of the results of their methods.

Supplementary Note 1

Why we prefer the NMWT method to the TMWT method. The NMWT method owns higher frequency-resolution than the TMWT method. The TMWT cannot distinguish two harmonic signals (i.e., the 6-year and 8.6-year signals) existing on the LOD intradecadal scales. Here, we will adopt a typical simulation example to demonstrate this point.

Simulation example: time span is defined as *t*=[1:1:1200] months, the sampling rate is defined as 1 month.

The simulated 6-year decaying signal is $y_1(t)$, see supplementary Fig.4a:

$$y_1(t) = 1.5e^{-0.00084t}\sin(2\pi f_1(t-18))$$

The simulated 8.6-year increasing signal is $y_2(t)$, see supplementary Fig.4b:

$$y_2(t) = 0.4e^{0.0008t}\sin(2\pi f_2(t-18))$$

where, f_1=0.01389cpm; f_2=0.00969cpm.

The composite signal (see supplementary Fig.4c) is as following

$$y(t) = y_1(t) + y_2(t)$$

Supplementary Fig.4d shows that the TMWT cannot separate the 6-year and 8.6-year signals (Supplementary Fig.4d), while NMWT can clearly recognize these two target harmonic signals with the different frequencies (Supplementary Fig.4e).

Supplementary Note 2

On the SSA method used to analyze the LOD data. We have further used SSA to analyze the LOD data. Through the following simulation analysis, we find that SSA method cannot well distinguish the intradecadal components existing in the LOD variations. As the previous works[10-11] indicated, the frequency-resolution of SSA is related to the window length parameter (L), and choosing an appropriate L value is important for SSA method to analyze the real series. As supplementary ref.10 indicated, L should neither be too large nor too small. If L is too small, the coarse resolution may cause several neighboring peaks in the spectrum of signal to appear as one, on the other hand, large L values will split the peak into several components with neighboring frequencies.

Here, we will use a simulation test and an actual LOD data to show the SSA results. We simulate a composite signal of the superposition of three harmonic components:

$$Y(t)=y_1(t)+y_2(t)+y_3(t)+\text{noise}(t)$$

where, the data length is 686 months, which is the same length as that of the currently observed LOD data (during 1962~2019); y_1, y_2 and y_3 are respective the 6-year (decaying), 7.2-year (steadying) and 8.6-year (increasing) signals, and their expressions are following:

$y_1=0.12\exp(-0.001t)\sin(2\pi f_1 t)$; $f_1=0.0138$cpm % a decaying 6yr signal

$y_2=0.05\sin(2\pi f_2 t)$; $f_2=0.01157$cpm % a steady 7.2yr signal

$y_3=0.05\exp(0.0009t)\sin(2\pi f_3 t)$; $f_3=0.00969$cpm % an increasing 8.6yr signal

The above $Y(t)$ series and the various corresponding SSA results are presented in supplementary Fig.5a. Furthermore, we show the SSA results in frequency domain, see supplementary Fig.5b. Here, we need to discuss how to choose the appropriate positive integral parameters of SSA method, i.e., L and I, where I reflects the so-called decomposition level, when I is set to be larger, the higher-frequency signal will be obtained, otherwise the opposite:

Firstly, if L is invariable (here L=600), then the SSA results (I equals to 1 alternative 2) can well characterize the above simulated composite signal- $Y(t)$, see the supplementary Fig.5a; when I ≥3, the SSA results tend to be non-ideal to characterize the target composite signal (supplementary Fig.5a). Consequently, we only need to use I=1 (or 2) to analyze the LOD intradecadal variations based on the SSA method. Meanwhile, supplementary Fig.5b indicates that the frequency-domain results of SSA method (with parameter I =1 and 2) present a wide energy-spectrum within the 5~10yr band rather than a 6-year sharp peak. Consequently, these SSA results cannot well distinguish the target signals (i.e., the 6-year, 7.2-year and 8.6-year) existing in the original composite signal.

Secondly, if I remains to be invariable (here I=2), when the parameter L is set to be relatively large (e.g., L=600,550), SSA results (supplementary Fig.6a) show a wide energy-spectrum (supplementary Fig.6b); when L is relatively small (e.g., 500, 450 and smaller), then SSA results will trend to be a single 6-year peak (supplementary Fig.6b), this corresponding time-domain result (supplementary Fig.6a) from SSA method, however, is inconsistent with the original simulated 6yr decaying oscillation. In addition, this single 6-year peak does not means that only a

6-year signal exists in the original composite series, since the original simulated series contains three frequency components.

Consequently, the above simulation analysis shows that although SSA method may well recover the original superposition signal $Y(t)$ when the parameter L an I are adopted to be the appropriate values (supplementary Fig.5a, supplementary Fig.6a, e.g., L=600, I=2), but it cannot effectively distinguish the above target 6-year, 7.2-year and 8.6-year components due to the frequency-resolution issue of the SSA method. The time domain result from SSA method (e.g., L=600, I=2) shows a modulation phenomenon, which actually attributes to the interaction of the various components within the 5~10yr band (supplementary Fig.5b, supplementary Fig.6b).

Furthermore, applying SSA method to the actual LOD intradecadal variations. According to the above simulation, we can use the relevant parameters (i.e., L=600,550,500,450, while I=2) to analyze the actual LOD intradecadal variations based on the SSA method, and the results (in both time and frequency domains) are shown in the supplementary Fig.7.

Comparing the supplementary Fig. 6 with the supplementary Fig.7, a wide energy spectrum range of LOD variations recovered by the SSA method is shown in supplementary Fig.7b (when L value is relatively larger, e.g., L=600 or 550), which shows that the actual LOD intradecadal variations should not contain a 6-year component alone. When L is adopted to be relatively smaller value (e.g., L=450), the spectrum of LOD variations recovered by the SSA method is shown to be a 6-year peak alone, which does not mean that the actual LOD variations only contains a 6-year oscillation alone within the 5~10year band. In summary, the SSA method is not an ideal approach to distinguish and accurately isolate the target intradecadal components existing in the LOD variations.

Supplementary Note 3

Why the NMWT method is suitable for extracting the target harmonic signal with the specific frequency?

The amplitude of the target signal presents a ridge line in the NMWT amplitude spectrum[5,12], we can recover the target signal through extracting the real coefficients in the NMWT spectrum corresponding to the locations of the ridge line, which can be seen as a line-pass filtering result[11,13] from the filter with the infinitely-narrow band-pass range in a certain sense. While the other works

to extract the LOD variations are usually based on the band-pass filter, the range of which is relatively wider and the target signal extracted by this filter will be easily disturbed by the other signals. For example, the 5~9 year variations of LOD in previous publications[4,14-15] was obtained by the band-pass filter, and the variations in this frequency-band cannot well characterize the time-varying features of the 6-year or the 8.6 year signal alone.

Supplementary Note 4

Simulation example 1

In order to make it easy for other researchers to repeat our data processing, we give the following specific expression of the background trend $T(t)$, which shows the saw-toothed change, see supplementary Fig.8.

$$T(t)=\begin{cases} \frac{3}{70}(t-1)+15 & t\in(1:350) \\ -\frac{2}{35}(t-350)+30 & t\in(351:700) \\ \frac{3}{20}(t-700)+10 & t\in(701:800) \\ -\frac{1}{10}(t-800)+25 & t\in(801:1000) \\ \frac{1}{40}(t-1000)+5 & t\in(1001:1200) \end{cases}$$

The simulated LOD variations is given by

$$S(t)=y_1(t)+y_2(t)+T(t)+noise(t)$$

where, the expressions of the $y_1(t)$ and $y_2(t)$ are shown in supplementary note 1; the noise(t) indicates the random noise signal.

We use the four steps (NMWT+ BEPME) proposed in the Methods to separately extract the 6-year signal and the 8.6-year signal, and the results are shown in the supplementary Fig. 9. This simulation example indicates that the results from the method (i.e., NMWT+BEPME) proposed in this work are generally well consistent with the original signals, especially the phase information are recovered perfectly. However, there are still some errors existing at the boundaries.

Additionally, we have also simulated the 8.6-year pure harmonic signal (see supplementary Fig. 10). Supplementary Fig.10 shows that if the 8.6-year signal existing in the LOD variations is a steady harmonic signal, then we should well recover it in the time domain, and its amplitude should be constant. This example further indirectly proves that the 8.6-year increasing signal found by this work is reliable.

Simulation example 2

Here, we further use the following typical simulation examples to test whether the intradecadal variations in LOD will be disturbed, as the background trend (period T >10.67yr) is removed, especially, to check whether the existence of the 8.6-year signal in LOD is the consequence of the removal of the background trend?

Given that a recent work[8] used the AR-z spectrum to analyze the LOD data during a longer time span (i.e., 1760-2018), which shows that there are various periodic components with periods longer than 10yr existing in the LOD data, such as ~149 yr, ~68 yr, ~33 yr, ~22.3 yr, ~18.6 yr, ~13.5 yr, ~11 yr. Assuming that the actual observed LOD variations does own so many signals, here we give the following simulation tests: The data length of this simulation analysis is set to be 686 months (~57yr), which is the same as that of the actual LOD data (i.e., 1962~2019), and the data sampling rate is defined as 1month. Here, the background trend is defined as the variations with period T >10 yr, and the expression of this simulated background trend (here, Trend for short) in the following part is written as

$$\text{Trend}(t)=h_1(t)+h_2(t)+h_3(t)+h_4(t)+h_5(t)+h_6(t)+h_7(t)=\sum_{i=1}^{7} h_i(t) \qquad (1)$$

where, t ~[0:685] and the time interval is 1month, $h_i(t)$ expresses the i th signal component, i.e., $h_i(t)=A_i\sin(2\pi f_i t)$; here, f_i signifies the frequency of the i th component, and A_i is the i th amplitude, and we can determine these A_i (i=1,2,3...7) values according to the relative amplitude values given by supplementary ref.8.

Furthermore,

f_1=1/(149×12) cpm; h_1=0.2sin(2πf_1 t); % 149yr periodic component

f_2=1/(68×12) cpm; h_2=0.26sin(2πf_2 t); % 68yr periodic component

f_3=1/(33×12) cpm; h_3=sin(2πf_3 t); % 33yr periodic component

f_4=1/(22.3×12) cpm; h_4=0.63sin(2πf_4 t); % 22.3yr periodic component

f_5=1/(18.6×12) cpm; h_5=0.2sin(2πf_5 t); % 18.6yr periodic component

f_6=1/(13.5×12) cpm; h_6=0.05sin(2πf_6 t); % 13.5yr periodic component

f_7=1/(11×12) cpm; h_7=0.06sin(2πf_7 t); % 11yr periodic component

where, cpm means cycles-per-month.

Constructing the following two simulated series

The first series: $Y(t)$ (i.e., Trend+8.6yr term+6yr term+noise term)

$$Y(t)=\text{Trend}+y1(t)+y2(t)+\text{noise}(t) \quad (2)$$

where, $y_1(t)$ and $y_2(t)$ are respective the 8.6yr term and 6yr term, noise (t) is a stochastic noise term.

And

$$y_1(t)=0.08\sin(2\pi f_8 t); \quad y_2(t)=0.12\sin(2\pi f_9 t)$$

here, f_8=1/(8.6×12) cpm, f_9=1/(6×12) cpm.

The second series: $H(t)$ (i.e., 8.6yr term +6 yr term)

$$H(t)=y1(t)+y2(t) \quad (3)$$

We make the following two types of comparisons

1) Comparison of the trend obtained by low-pass filter (the green curve in supplementary Fig. 11a) and the simulated original background Trend (the red curve in supplementary Fig.11a);
2) Comparison of the residual series obtained by removing the background trend (the blue curve in supplementary Fig.11b) and the above $H(t)$ (the purple curve in supplementary Fig. 11b).

Supplementary Fig. 12 shows the comparison of the NMWT time-frequency spectrum of the $H(t)$ signal and the residual series after removing the background trend, while supplementary Fig.13 indicates the comparison of the Fourier spectrum between the residual series and $H(t)$. The above simulation results (supplementary Figs.11-13) show that the results after removing the background trend are general well consistent with the original intradecadal variations $H(t)$ (i.e., the 6yr+8.6yr).

Of course, according to the above test results (supplementary Figs.11-13), we cannot

guarantee that the residual series obtained in this work must be accurate completely, but we can demonstrate that this result is accurate enough in general. In addition, we would like to argue that this work focuses on the LOD data analysis in both time and frequency domain (Fig. 1), which differs from the previous work[2]. Moreover, the time-frequency domain results (Fig. 1b,c,d) show that the LOD intradecadal variations with a wide energy-spectrum range present the existence of the different frequency components. Consequently, from the perspective of time-frequency joint analysis, this work not only presents a 6-year oscillation, but also shows an 8.6-year signal and its time-varying feature.

Conversely, if the intradecadal variations only contain a single 6-year oscillation with an almost constant amplitude as suggested by previous work[2], then the Fourier spectrum should present a sharp peak. However the actual LOD intradecadal variations in frequency domain does not support this case (see the blue curve in Fig. 1b). That is to say, the actual LOD intradecadal variations should own other frequency components besides the 6-year component, moreover, NMWT spectrum can clearly present the existence of an 8.6-year oscillation.

Here, we further test whether the target harmonic signals (e.g., 6yr term and 8.6yr term) are disturbed by removing the background trend? Considering the following composite signal

$$Y(t)= \text{Trend(t)}+8.6\text{yr term}+6\text{yr term} +\text{noise term}$$

where, the two intradecadal terms (i.e., 6-year and 8.6-year) are simulated respectively through the following three cases

1) Amplitude steady signals, 2) Amplitude decaying signals, 3) Amplitude increasing signals

Simulation test 1: **Recovery of the 6yr oscillation in terms of the three cases**

In our work, how to recover the above three types of 6yr oscillation signals from the above simulated composite signal- $Y(t)$? In each case, we perform the following steps:

Firstly, we use the wavelet low-pass filter mentioned above to obtain and remove the background trend from $Y(t)$ series;

Secondly, we obtain the residual series;

Finally, we use the NMWT+BEPME method to recover the target 6yr signal from the residual series, and the results are shown in supplementary Fig. 14.

Simulation test 2: **Recovery of the 8.6yr oscillation in terms of the three cases**

How to recover the three types of the 8.6yr oscillation signals from the above simulated composite signal- $Y(t)$? the data processing steps are the same as that of the 6yr oscillation recovery mentioned above. And the results are shown in supplementary Fig. 15.

Supplementary Note 5

Simulation tests of the AR-2 damped stochastic oscillation model

1) A single stochastic damped oscillation case

Formula (6) in the Methods of this work indicates that the time-varying characteristic of $y(t)$ essentially depends on the specific form of $E(t)$. If $E(t)$ is a stochastic white noise series, see supplementary Fig.16a, then we can generate a forced damped oscillation series on the basis of formula (6) (here β=8.4×10^{-4}/month in supplementary Fig.16b from the supplementary ref.7), the result of which is shown in supplementary Fig.16c, which shows that the 6-year oscillation presents a modulated amplitude changes under the action of a stochastic excitation. Furthermore, we apply the NMWT method to the damped 6-year stochastic oscillation series, and the result is displayed in supplementary Fig.17, which indicates that there is still only one obvious 6-year periodic component existing in the NMWT spectrum.

In this test, we repeatedly use different random excitation sequences $E(t)$ as the input series to simulate a variety of output results, see supplementary Fig. 18. Based on our repeated simulation tests, in some cases, the peak splitting phenomena appear in the NMWT spectrum, e.g., the output 6 and output 9 shown in supplementary Fig.18. However, we find that even though a monochromatic signal (e.g., the 6-year oscillation normal mode signal in LOD) presents a modulated phenomenon under the action of an arbitrary continuous stochastic excitation and its time-frequency spectrum may perform the similar behaviors as the case of output 6 presented in supplementary Fig.18, the energy and the width of the spectral bands of these splitting components in NMWT spectrum are still small and narrow.

2) The case of two stochastic damped oscillation simulations

If there are two signals (i.e., 6-year and 8.6-year) that are respectively convoluted by two different continuously stochastic series, and the cases of which are simulated to be presented in the

supplementary Fig.19. NMWT method also can provide a good recovered result (including the decaying information, increasing information, the correct phase information) of the target monochromatic signals from the original data series. Here, according to the simulation test (e.g., the output 9 series) presented in supplementary Fig.18, we do not exclude the possibility that the weak signals with periods ranging from 6-year to 8.6-year presented in the LOD spectrum may be the consequence of the stochastic excitation of the two free normal mode signals (e.g., the 6-year and 8.6-year oscillations). Of course, the more definitive conclusion still needs to be further studied.

Supplementary Note 6

Further discussion about the results from NMWT+BEPME method

The result recovered by our method (i.e., NMWT+BEPME) used in this manuscript actually depends on the time-varying characteristic of the original oscillation signal. That is to say, if a 6yr decaying oscillation signal (here, the decaying information is unknown in advance) exists in the original observed series (e.g., the observed LOD data), then the result recovered by NMWT+BEPME method will be a decreasing signal as well. Nevertheless, a periodic decreasing oscillation signal may include two cases: 1) a free damping oscillation (i.e., the theoretical oscillation curve, see supplementary Fig.16b), 2) the consequence of a continuous stochastic excitation (see the data during 1000~2000 months in supplementary Fig.16c). If it is just the former case, then we can nicely recover the real free damped oscillation (in this case, the observed quality factor Q value equals to the theoretical value, where the Q value estimated from the damping oscillation result recovered by the NMWT+BEPME is called as the observed Q value), we have demonstrated this point in our previous works (e.g., Duan et al,2017; Duan and Huang 2019, and also in this work). However, if it is the latter case, then the output series via the continuous stochastic excitation may be various with the different input series (e.g., supplementary Fig.18), that is to say, if the output result (or the original observed signal) is decaying, then the result recovered by our method will be decreasing as well, nevertheless, at this time, the wavelet result may not reflect the free damping oscillation, which is similar to the Chandler wobble (CW) variations in the polar motion, and in this case, the observed Q value may be no longer its theoretical Q value, therefore, in this case, the decaying oscillation recovered by

the NMWT method does not necessarily reflect the actual free damped oscillation.

Despite this, we would like to mention the advantage of NMWT method once again: even though we do not know the prior information (e.g., the decaying or increasing) of the target signals in advance, we still can recognize this decaying (or increasing) signal and recover it perfectly in the time domain[13] through using the NMWT+BEPME method. As supplementary ref.7 indicated, if the observed 6yr oscillation in LOD is consequence of the significantly continuous stochastic excitation, then the current Q ~51 from supplementary ref.7 may be not the theoretical value of the 6-year free normal mode signal. Therefore, we call this value Q (~51) as the observed quality factor in this work (of course, also in supplementary ref.16), which is similar to the CW. Of course, as supplementary ref.7 discussed, we agree with the idea that the observed 6-year oscillation should be the result of excitation, however, the question is that, up to now, we have not found the strong evidence to demonstrate that the observed 6-year oscillation in LOD must be subject to the significantly continual stochastic excitation during 1962~2019, since, up to now, we have not found the reliable stochastic excitation series (or excitation events). Conversely, the 6-year oscillation time-domain result recovered by the NMWT method can be nicely characterized by a typical exponential decaying function (see supplementary ref.16), furthermore, the strength of electromagnetic coupling (i.e., ~38$Nm^{-3}s$) at the core-mantle boundary inferred by this observed quality factor value (i.e., Q ~51) is also acceptable[14], so we gave a possible explanation that the current 6-year signal decaying during the past several decades (i.e., 1962~2012) detected by supplementary ref.5 is due to the electromagnetic coupling dissipation at the core-mantle boundary[7,16-17].

As to the two damped oscillation cases: If these two oscillation signals are free decaying oscillations, then NMWT+BEPME can well recover them respectively in the time domain[13]. Furthermore, the above simulation tests have shown that NMWT method owns a high frequency-resolution, which can clearly distinguish the 6yr and 8.6yr components. If these two signals are the damped stochastic oscillations which are presented in the supplementary Fig.19, we can recover these decaying information of the original signals using NMWT+BEPME method and further use these recovered attenuated results to further infer the relevant excitation information. In summary, combining the NMWT method with the BEPME strategy is indeed an effective harmonic analysis approach to obtain a good recovered result (including the decaying or

increasing information, the correct phase information) of the target monochromatic signal from the original data series (e.g., see the supplementary Figs. 14-15).

Supplementary References

1. Abarco del Rio, R., Gambis D. & Salstein, D.A. Interannual signals in length of day and atmospheric Angular momentum. Ann Geophys. 18,347-364 (2000).
2. Holme, R. & de Viron, O.Characterization and implications of intradecadal variations in length of day. Nature. 499, 202-205 (2013).
3. Chao, B.F., Chung, W.Y., Zong, R., Shih. & Hsieh, Y.K.. Earth's rotation variations:a wavelet analysis. Terra. Nova. 26, 260-264 (2014).
4. Gillet, N., Jault, D. & Finlay, C. C. Planetary gyre, time-dependent eddies, torsional waves and Equatorial jets at the Earth's core surface, J. Geophys. Res: Solid Earth. 120, 3991-4013, doi:10.1002/ 2014JB011786 (2015).
5. Duan, P.S., Liu, G.Y., Liu, L.T., Hu, X.G., Hao, X.G., Huang, Y., Zhang, Z.M. & Wang, B.B. Recovery of the 6 year signal in length of day and its long term decreasing trend. Earth Planets Space. 67:161, doi:10.1186/s 40623-015-0328-6 (2015).
6. Stephenson, F., Morrison, L. & Hohenkerk, C. Measurement of the Earth's rotation: 720 BC to AD 2015, Proc. R. Soc. A, 472, 20160404, doi:10.1098/rspa.2016.0404 (2016).
7. Duan, P.S., Liu, G.Y, Hu, X.G, Sun, Y.F. & Li, H.L. Possible damping model of the 6 year oscillation signal in length of day. Phys. Earth Planet. Inter, 265. 35-42 (2017).
8. Ding, H. Attenuation and excitation of the ~6 year oscillation in the length-of-day variation. Earth Planet. Sci. Lett., 507,131-139 (2019).
9. Ding, H. & Chao, B.F. A 6-year westward rotary motion in the Earth: Detection and possible MICG coupling mechanism. Earth Planet. Sci. Lett. 495, 50-55 (2018).
10. Chen, Q., Van Dam, T., Sneeuw, N., Collilieux,X., Weigelt, M. &Rebischung, P. Singular spectrum analysis for modeling seasonal signals from GPS time series. J. Geodyn. 72, 25-35 (2013).

11. Wang, G.C., Liu, L.T., Xu, A.G., Pan, F., Cai, Z.W., Xiao, S.H., Tu, Y. &Li, Z.H. On the capabilities of the inaction method for extracting the periodic components from GPS clock data. GPS. Solution, 22, 92 (2018).

12. Liu, L.T., Hsu, H.T. & Grafarend, E.W. Normal Morlet wavelet transform and its application to the Earth's polar motion. J. Geophys. Res. 112, B08401,doi10.1029/2006JB004895 (2007).

13. Duan, P. S.& Huang, C. L. Application of normal Morlet wavelet transform method to the damped harmonic analysis: On the isolation of the seismic normal modes (${}_0S_0$ and ${}_0S_5$) in time domain. Phys. Earth Planet. Inter, 288, 26-36 (2019).

14. Gillet, N., Jault, D., Canet, E. & Fournier, A. Fast torsional waves and strong magnetic field within the Earth's core. Nature. 456, 74-77 (2010).

15. Gillet, N., Huder, L.& Aubert, J. A reduced stochastic model of core surface dynamics based on geodynamo simulations. Geophys. J. Int. 219(1), 522-539 (2019).

16. Duan P S, Liu G Y, Hu X G, Zhao, J. & Huang C L. Mechanism of the interannual oscillation in length of day and its constraints on the electromagnetic coupling at the core-mantle boundary. Earth Planet. Sci. Lett. 482, 245-252 (2018).

17. Duan, P. S. & Huang, C. L. On the mantle-inner core gravitational oscillation under the action of the electromagnetic coupling effects. J. Geophys. Res: Solid Earth. 125, e2019JB018863.https:/doi/.org/10.1029 /2019JB018863 (2020).
